# Are Eyes a Mirror of the Soul? What Eye Wrinkles Reveal about a Horse’s Emotional State

**DOI:** 10.1371/journal.pone.0164017

**Published:** 2016-10-12

**Authors:** Sara Hintze, Samantha Smith, Antonia Patt, Iris Bachmann, Hanno Würbel

**Affiliations:** 1 Division of Animal Welfare, Vetsuisse Faculty, University of Bern, Bern, Switzerland; 2 Agroscope, Swiss National Stud Farm, Avenches, Switzerland; 3 Royal (Dick) School of Veterinary Studies, University of Edinburgh, Edinburgh, United Kingdom; 4 Department of Animal and Avian Sciences, University of Maryland, College Park, Maryland, United States of America; The University of Melbourne, AUSTRALIA

## Abstract

Finding valid indicators of emotional states is one of the biggest challenges in animal welfare science. Here, we investigated in horses whether variation in the expression of eye wrinkles caused by contraction of the inner eyebrow raiser reflects emotional valence. By confronting horses with positive and negative conditions, we aimed to induce positive and negative emotional states, hypothesising that positive emotions would reduce whereas negative emotions would increase eye wrinkle expression. Sixteen horses were individually exposed in a balanced order to two positive (grooming, food anticipation) and two negative conditions (food competition, waving a plastic bag). Each condition lasted for 60 seconds and was preceded by a 60 second control phase. Throughout both phases, pictures of the eyes were taken, and for each horse four pictures per condition and phase were randomly selected. Pictures were scored in random order and by two experimenters blind to condition and phase for six outcome measures: qualitative impression, eyelid shape, markedness of the wrinkles, presence of eye white, number of wrinkles, and the angle between the line through the eyeball and the highest wrinkle. The angle decreased during grooming and increased during food competition compared to control phases, whereas the two phases did not differ during food anticipation and the plastic bag condition. No effects on the other outcome measures were detected. Taken together, we have defined a set of measures to assess eye wrinkle expression reliably, of which one measure was affected by the conditions the horses were exposed to. Variation in eye wrinkle expression might provide valuable information on horse welfare but further validation of specific measures across different conditions is needed.

## Introduction

Animal welfare comprises both physical health and emotional well-being. Whereas physical health can be assessed directly, the subjective experience of emotions must be inferred [1]. Ideally, indicators of emotional state are non-invasive and can be assessed easily and reliably in various contexts.

Charles Darwin already observed that ‘man and animals express the same state of mind by the same movements’ ([2], p.352), highlighting facial expressions as potential indicators of animal emotions. However, while studied extensively in humans (e.g. [3,4]) and non-human primates (e.g. [5]), facial expressions have been largely neglected in other species until more recently (e.g. [6,7]).

In animals, facial expressions have mainly been investigated using Facial Action Coding Systems (FACS) and Grimace Scales (GS). FACS was originally developed in humans [3] and relies on the systematic characterisation of facial expression based on individual Action Units which are caused by contraction or relaxation of one or more muscles. It has been proposed as a tool to assess pain and distress in neonates and infants who cannot express their feelings verbally [8], as well as for the diagnosis of depression [9]. Based on the approach in humans, FACS have now been developed for non-human animals, including various primates (e.g. ChimpFACS [5], OrangFACS [10]), dogs (DogFACS [11]), and horses (EquiFACS [12]). GS for pain assessment have been developed and validated by comparing facial expressions in painful and painless conditions in mice (MouseGS [7]), rats (RatGS [13]), rabbits (RabbitGS [6]), cats [14], sheep (Sheep Pain Facial Expression Scale (SPFES) [15]), and horses (HorseGS [16], Equine Pain Face [17]).

In horses, two independent studies were performed in which pain was induced by either surgery [16] or ischemic pain and neurogenic inflammation [17], from which the HorseGS [16] and the Equine Pain Face [17] were developed. More recently, a FACS for horses (EquiFACS) has been established which describes all possible facial movements [12]; however, it has not been applied to specific emotional contexts (e.g. pain) so far.

One particular aspect of facial expression in horses is the formation of wrinkles above the eyeball. Eye wrinkles are caused by contraction of the inner eyebrow raiser (levator anguli oculi medialis muscle and corrugator supercilii muscle) and have been identified as an Action Unit (AU 101) in EquiFACS [12]. Among horse owners, eye wrinkles are also known as ‘worry wrinkles’, suggesting that they may be associated with a negative emotional state. Furthermore, eye wrinkles were more pronounced in the pain treatment as assessed by the Equine Pain Face [17], and in the Horse GS a ‘tension above the eye area’ was described in horses that were in pain ([16], p.4). Thus, present evidence indicates that pain may be associated with eye wrinkles. Whether the expression of eye wrinkles may serve more generally as a sign of negative emotional states, has not been studied so far. Supporting evidence stems from studies in humans, where it was found that the same Action Unit associated with eye wrinkles in horses was associated with fear and sadness in people [18]. Based on this, we aimed at examining the link between emotional state and eye wrinkle expression in horses.

For this purpose, we exposed horses to distinctively valenced conditions. Since neither EquiFACS nor the Horse GS have looked at eye wrinkle expression in more detail than describing the presence or absence of the wrinkles, we established a range of outcome measures that can be scored reliably to assess variation in eye wrinkle expression. We hypothesised that positive emotions would reduce the expression of eye wrinkles whereas negative emotions would increase it.

## Materials and Methods

### Animals and housing

This study included 16 horses (15 stallions, 1 mare) of three breeds (Franches-Montagnes, warmblood, trotter) aged between 3 and 20 years (10.4 ± 4.7, Table 1). The sample was randomly selected from the horse population of the stud farm, excluding subjects that were not responding to food competition during pilot observations (see below). All horses were housed in standard single boxes (3.00 x 3.50 m) at the Swiss National Stud Farm of Agroscope (Avenches, Switzerland). The stable was divided into six discrete areas. Each area contained eight horse boxes in two rows separated by an aisle (3 m). All boxes had a window facing into the adjacent aisle. The 16 experimental horses were distributed across each of the six areas, with a maximum of four horses from the same area. They were kept on wood shavings or straw and had visual contact with conspecifics. Horses were fed hay and concentrate three times a day, and water was provided ad libitum by automatic drinkers. On a daily basis, horses were exercised (riding, carriage riding), allowed free movement on a sand paddock, or walked in a horse walker, according to the Swiss Animal Welfare Ordinance [19].

**Table 1:** Sex, age and breed of the 16 horses used in this study.

Sex	Year of birth	Breed
Stallion	2011	Franches-Montagnes
Stallion	2010	Warmblood
Stallion	2008	Warmblood
Stallion	2006	Franches-Montagnes
Stallion	2005	Franches-Montagnes
Stallion	2005	French Trotter
Stallion	2004	Franches-Montagnes
Stallion	2003	Franches-Montagnes
Stallion	2003	Franches-Montagnes
Stallion	2002	Franches-Montagnes
Stallion	2002	Franches-Montagnes
Stallion	2000	Franches-Montagnes
Stallion	1999	Franches-Montagnes
Stallion	1997	Franches-Montagnes
Stallion	1994	Franches-Montagnes
Mare	2009	Warmblood

### Experimental design

Each horse was exposed to four conditions, two positive (grooming, food anticipation) and two negative (food competition, waving a plastic bag).

Each condition was divided in two phases lasting 60 seconds each: control phase followed by treatment phase. Food anticipation, food competition, and waving of a plastic bag did not involve the presence of an experimenter inside the box, whereas grooming was performed by the experimenter standing next to the horse inside the box. Consequently, the experimenter was also present during the control phase of the grooming condition, imitating the movement of grooming beside the horse without touching it. Throughout the whole duration (= 60 seconds) of both treatment and control phase, pictures of the eyes were taken by two professional photographers (camera: Canon EOS-1D X, Switzerland; lens: EF 70–200 mm f/.8 L IS II USM, Switzerland) who were positioned in the aisle. Horses were also exposed to all stimuli but grooming from the side of the aisle.

A total of ten experimental sessions was conducted during May and June 2014 with five to eight horses being exposed to one condition per session. Sessions took place during off work-hours (between 11:45 and 13:30) to avoid disruption of the horses’ attention and emotional changes caused by management procedures. During all sessions the same two experimenters and two photographers were present. All 16 horses were exposed to all four conditions, with the order of the conditions being counterbalanced between horses. No more than two conditions per session were presented in the same area of the stable, with the positive conditions (grooming, food anticipation) always preceding the negative conditions (food competition, plastic bag) because the positive conditions affected only the experimental horse, whereas negative conditions could affect all horses within the same area. In one session, it was unavoidable to expose two horses within the same area to negative conditions; however, food competition, a common condition for the horses, preceded the plastic bag condition by nearly 90 minutes, thereby minimising the risk of contamination.

Before the start of each session, the forelocks of the horses were braided to ensure clear view of both eyes. Lights were switched on and two additional flood lights were arranged to improve the illumination of the boxes. Horses were habituated to the flood lights before the start of the experiment. Habituation was deemed successful when horses did not react to the flood lights anymore. Windows heading to the aisle were opened to allow the horses to put their heads out during testing.

### Conditions of different emotional valence

#### Grooming (G)

Grooming has been shown to reduce heart rate in horses [20,21] which is indicative of a more relaxed state and to induce positive behavioural responses, like leaning into the massage [21]. In the present experiment, horses were groomed continuously for 60 seconds by a familiar experimenter (SH) standing on the left side of the horses and moving the fingertips of both hands along the withers, the shoulder and the neck. Grooming was preceded by a 60 second control phase with the experimenter imitating the grooming movements without touching the horse.

#### Food anticipation (FA)

Anticipation is a motivational state between a cue signaling the arrival of an event and the actual arrival of this event [22,23]. In order to induce a positive anticipatory state, horses were conditioned to anticipate a special food reward (British Mash Original, Landmühle, Switzerland, mixed with apples and carrots) when confronted with a blue and yellow striped bucket for three trials across two days preceding the test day. During conditioning as well as on the test day, the bucket was shown to the experimental horse, and after a 60 second waiting period (= anticipation and therefore treatment phase during the experiment), the horse was given access to the bucket with the food. Horses were trained individually and with a bucket and food different from their usual food to distinguish clearly between food anticipation and food competition. The 60 seconds preceding presentation of the bucket were used as control phase.

#### Food competition (FC)

Witnessing other horses being fed their regular rations of concentrates while waiting for being fed is stressful for stabled horses [24]. From our pilot observations we knew that most horses at the stud farm respond strongly to their neighbours being fed, for example, by shaking the head quickly up and down, flattening the ears, and threatening the neighbour. We used the common procedure of feeding concentrate to the neighbour horses to induce frustration in the experimental horse. Moreover, instead of horses being fed between 11:00 and 11:30 by the farm workers, feeding was delayed by about one hour to increase frustration [25]. Although both control and treatment phase were affected by this delay in feeding, we expected a larger contrast due to enhanced frustration during the treatment phase. One of the experimenters entered the aisle with a food trolley, feeding all horses with the experimental horse being fed last and after a minimum of 60 seconds. The 60 seconds preceding the experimenter entering the aisle with the food trolley were used as control phase.

#### Plastic bag (PB)

Waving a plastic bag has previously been used to induce fear in horses [26,27]. Similar to these studies, a plastic bag filled with cans and tied to the end of a whip and a green umbrella were used to startle the horses. The objects were waved in front of the box throughout the 60 seconds treatment phase. The 60 seconds preceding the waving period were used as control phase. To minimise the risk of habituation to this stimulus, only one horse per area was exposed to the plastic bag within one session. Moreover, whenever possible, other experimental horses of the same area were brought to outside paddocks before the start of the session.

### Picture processing

All pictures were screened and blurry pictures and pictures with obscured view of the eye wrinkles were excluded. Pictures were defined as blurry if eye wrinkles were not clearly visible or the beginning or end of wrinkles was not clearly detectable. The view of eye wrinkles was considered as obscured if either both eyes were visible (frontal picture) or if only parts of the eyeball were visible (frontal picture or picture from the back).

From the remaining pictures, four pictures per condition and phase were selected randomly for each horse using a random number generator in R (version 3.1.3, function ‘sample’, excluding repetitions), resulting in a total of 512 pictures (16 horses x 4 conditions x 2 phases x 4 pictures). Selected pictures were cropped and rotated with Adobe Lightroom 5.4 to make eye size and eye position comparable between pictures

### Outcome measures

**Fig 1:**
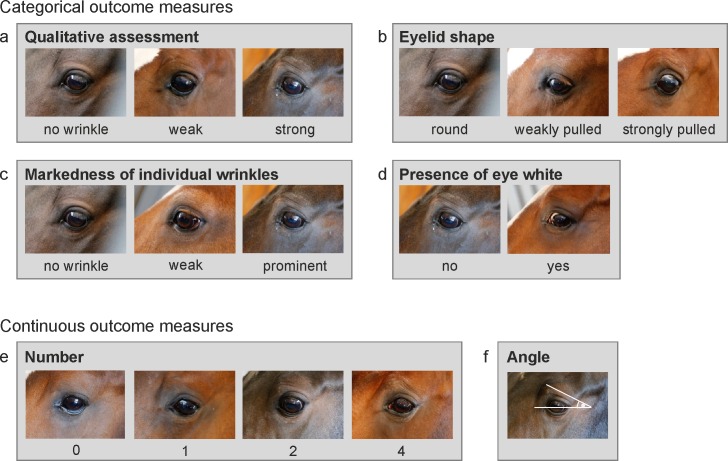
Measures for the assessment of eye wrinkle expression. (a) Qualitative assessment: Categories reflect the first subjective impression of the expression of eye wrinkles based on the number of wrinkles, their markedness, and the angle they are forming. ‘No wrinkle’: no wrinkle visible. ‘Weak’: the overall impression of the wrinkles is weak with, e.g., some weakly marked wrinkles forming a narrow angle. ‘Strong’: the overall impression of the wrinkles is strong with, e.g., several marked wrinkles forming a wide angle above the eyeball. (b) Eyelid shape: ‘Round’: smooth curve without any sign of the lid being pulled in the dorso-medial direction. ‘Weakly pulled’: curve is continuous but slightly pulled in the dorso-medial direction. The eye looks more angled. ‘Strongly pulled’: the lateral part of the lid is an almost straight line. (c) Markedness: the depth and width of the wrinkles is assessed. If the markedness differs between wrinkles, the most prominent wrinkle is assessed. ‘No wrinkle’: no wrinkle visible. ‘Weak’: wrinkles are flat and narrow lines. ‘Strong’: wrinkles are pronounced in depth and width. (d) Eye white: The sclera (eye white, also when brownish due to pigments) is assessed as visible (‘yes’) or not visible (‘no’). (e) Number: Only wrinkles above the eyelid and those of a minimum length of one third of the eyeball’s diameter are considered. A deep indent, often seen in older horses, is not considered as a wrinkle (as it is not caused by muscle contraction of the inner eyebrow raiser). Moreover, wrinkles originating on the eyelid (mostly one or two) are not counted. (f) Angle: The degree of the angle is measured on the intersection of the extension of a line drawn through the eyeball and the extension of the highest wrinkle. The line through the eyeball extends from the medial to the lateral corner of the eyeball. If the medial corner is not clearly defined, the line goes through the middle of the tear duct.

Outcome measures were defined based on pilot observations during which pictures of horses varying in eye wrinkle expression were compared in view of identifying those parts that differed between pictures, similar to the selection process of Actions Units in the HorseGS [16]. We defined six measures including four categorical measures: qualitative assessment (‘qualitative’), eyelid shape (‘shape’), markedness of eye wrinkles (‘markedness’), and presence of eye white (‘eye white’), as well as two continuous measures: number of wrinkles (‘number’), and angle (‘angle’). The definitions of the outcome measures and the details of scoring are specified in Fig 1.

### Scoring

All pictures were scored using CorelDRAW Graphics Suite X7 (Corel Corp.). The order of the pictures was randomised and the coder (SH) was blind to condition and phase. After every 50^th^ picture, ten out of these 50 pictures (i.e. 20%) were randomly chosen using the sample function in R and re-scored to assess intra-observer reliability. Additionally, 10% of the total sample were scored by a second coder to test for inter-observer reliability. The second coder was trained with example pictures before coding the sample.

### Ethical considerations

This experiment was carried out in accordance with the ethical policy of the International Society for Applied Ethology. It was approved by the Cantonal Veterinary Office according to the Swiss Animal Welfare legislation (Vaud, Switzerland, Approval Number VD 2804).

### Statistical analyses

#### Intra- and inter-observer reliability

Intra- and inter-observer reliability were analysed using SPSS (version 22). For categorical variables (‘qualitative’, ‘shape’, ‘markedness’, and ‘eye white’), Cohen’s kappa was calculated, with a significant p-value and a kappa-value above 0.8 indicating ‘almost perfect agreement’ [28]. Reliability of continuous measures (‘number’, ‘angle’) was assessed by intraclass correlation coefficients (ICC). ICCs were calculated using a two-way mixed design to assess the absolute agreement between the two scores [29,30], with an ICC greater than 0.75 indicating ‘excellent’ agreement [31]. Lower bounds of the 95% confidence interval (CI) were assessed as a measure of deviation from the ICC.

#### Assessment of the outcome measures

(Generalised) linear mixed-effects models and ordered logistic regressions were used for the analysis of the outcome measures to adequately reflect dependencies in the experimental design (nesting, repeated measures). Data were analysed in R (version 3.1.3).

All models were built based on the hypothesis that the two positive treatment phases would decrease eye wrinkle expression whereas the two negative treatment phases would increase it compared to the respective control phases. Thus, we expected an interaction between ‘condition’ (factor with four levels: grooming (G), food anticipation (FA), food competition (FC), plastic bag (PB)) and ‘phase’ (factor with two levels: control, treatment) to have an effect on the outcome measures. Since the position of the horse within its box was unpredictable during data collection, the side of the eye (left eye, right eye) varied among pictures. For each horse, condition, and phase, we only compared pictures of the same eye with each other. If a condition for a horse included pictures of both the left and the right eye, we excluded the pictures of the side containing fewer pictures. Further, ‘side’ was included as an additional fixed effect in all models to control for potential laterality effects (effect between horses).

Condition, phase, side, all two-way interactions (between condition and phase, condition and side, and phase and side) and the three-way interaction (condition, phase, and side) were implemented as fixed effects in all models. Random effects for all models were phase nested in condition nested in horse. All six measures described as outcome measures were considered as primary outcome variables (see above and Fig 1). The threshold of significance was adjusted to a p-value of p < 0.0083 using the Bonferroni correction for multiple hypothesis testing in order to control for type I error (false positive) rate.

In cases of significant interactions between condition and phase, Bonferroni-corrected post-hoc analyses were conducted (function: glht, package: multcomp).

Model assumptions were verified by graphical inspections of the residuals. We checked for normal distribution and homogeneity of variance for the linear models, and for homogeneity of variance for the generalised model and the ordered logistic regression. Transformation of the data was not necessary for any of the models.

**Categorical measures:** The binomial outcome measure ‘eye white’ was analysed using a generalised linear mixed-effects model (function: glmer, package: lme4 [32]). The multinomial outcome measures ‘qualitative’, ‘shape’ and ‘markedness’ were scored across three ordered levels each, and were thus analysed using an ordered logistic regression (function: clmm, package: ordinal [33]).

**Continuous measures:** For the continuous outcome measures ‘number’ and ‘angle’, linear mixed-effects models were run (function: lme, package: nlme [34]). For ‘angle’, pictures were only included in the analysis if a wrinkle was present such that an angle could be measured. Therefore, the sample for the outcome measure ‘angle’ consisted of all pictures with ‘number’ ≥ 1.

## Results

### Sample size and data structure

Based on the study design, we aimed at a total of 512 pictures (16 horses x 4 conditions x 2 phases x 4 pictures) for the scoring of eye wrinkles. However, the final sample of pictures meeting our inclusion criteria comprised 417 pictures. Some pictures included in the final sample were not good enough for assessing all outcome measures reliably. Thus, missing values occurred for the measures ‘shape’ (n = 11), ‘markedness’ (n = 6), ‘number’ (n = 49), and ‘angle’ (n = 31), but not for ‘qualitative’ and ‘eye white’. ‘Angle’ could only be analysed when there was at least one wrinkle (‘number’ ≥ 1), leading to a final sample for the analysis of ‘angle’ of 227 pictures.

Among the final sample, both levels of ‘eye white’ and all three levels of ‘shape’, ‘markedness’, and ‘qualitative’ were represented, and the ‘number’ of wrinkles ranged from 0 to 6 wrinkles, whereas ‘angle’ varied between 10.3° and 42.5°.

Pictures were fairly equally distributed across eye side, with 197 pictures of the left eye and 220 pictures of the right eye, and eye side was fairly well balanced across horses and conditions (S1 Table).

### Intra-observer reliability

Comparison of the first and the second scoring revealed high intra-observer reliability for all six outcome measures. For the categorical measures, all p-values were highly significant (p < 0.001) and all kappa-values were above 0.8 (‘qualitative’: κ = 0.856, ‘shape’: κ = 0.823, ‘markedness’: κ = 0.841, ‘eye white’: κ = 0.978). For both continuous measures, ICCs revealed excellent agreement between the two scores (‘number’: ICC_average_ = 0.976, CI lower bound = 0.963; ‘angle’: ICC_average_ = 0.979, CI lower bound = 0.968).

### Inter-observer reliability

Comparison of the scorings from the two coders revealed ‘almost perfect’ agreement for the categorical measures, ‘qualitative’, ‘markedness’ and ‘eye white’ (‘qualitative’: κ = 0.810, ‘markedness’: κ = 0.847, ‘eye white’: κ = 0.839), whereas agreement was moderate for ‘shape’ (κ = 0.497). P-values for all categorical measures were highly significant (p < 0.001). The agreement for the two continuous measures was ‘excellent’ (‘number’: ICC_average_ = 0.970, CI lower bound = 0.934; ‘angle’: ICC_average_ = 0.947, CI lower bound = 0.902).

### Assessment of the outcome measures

**Fig 2:**
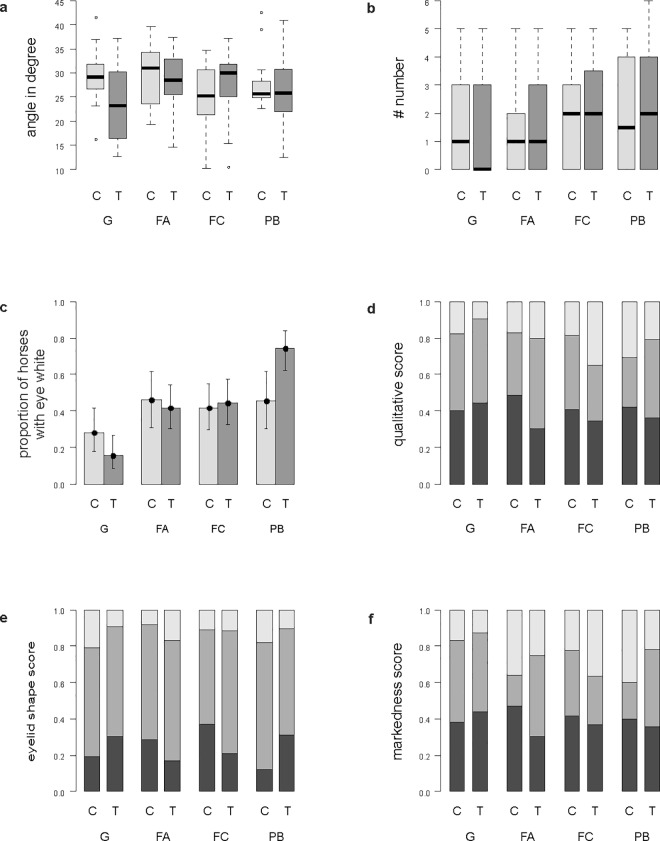
Effect of the four conditions on eye wrinkle expression. The effect of the four conditions (Grooming (G), Food anticipation (FA), Food competition (FC), Plastic bag (PB)) on the six outcome measures is shown with respect to phase (Control (C), Treatment (T)). (a, b) ‘angle’, ‘number’: boxplots with median (black line), interquartile range (box), 1.5 x interquartile range (whiskers). (c) ‘eye white’: bar chart±binomial confidence interval. (d, e, f) ‘qualitative assessment’, ‘eyelid shape’, ‘markedness’: stacked bar charts with the three colours representing the three different scores. (d) ‘qualitative assessment’: ‘no wrinkle’ (black), ‘weak’ (dark grey), ‘strong’ (light grey). (e) ‘eyelid shape’: ‘round’ (black), ‘weakly pulled’ (dark grey), ‘strongly pulled’ (light grey). (f) ‘markedness’: ‘no wrinkle’ (black), ‘weak’ (dark grey), ‘strong’ (light grey).

‘Angle’ was significantly affected by the interaction between condition and phase (F_3_,_22_ = 5.922, p = 0.004, Table 2). Post-hoc comparisons revealed a significant difference between control and treatment phase in the G condition (z = -3.258, p = 0.008) and in the FC condition (z = 2.803, p = 0.033). As predicted by our hypothesis, ‘angle’ decreased from the control to the treatment phase during the G condition and increased from the control to the treatment phase during the FC condition (Fig 2). However, there was no significant change between control and treatment phase during the FA and the PB conditions. There was also a non-significant interaction for ‘angle’ between condition and side but independent of phase, indicating the possibility of laterality effects (F_3,146_ = 3.511, p = 0.02, Table 2). The control phases did not significantly differ from each other (F_3,15_ = 1.732, p = 0.2).

**Table 2:** Statistical models and results for the six outcome measures.

Outcome measure	Model	Interaction	Test statistic	P-value ^1^
Qualitative assessment	Ordered logistic regression	condition x phase x side	χ^2^_3_ = 3.795	0.28
		condition x phase	χ^2^_3_ = 3.902	0.27
		condition x side	χ^2^_3_ = 2.568	0.46
		phase x side	χ^2^_1_ = 3.095	0.08
Eyelid shape	Ordered logistic regression	condition x phase x side	χ^2^_3_ = 2.008	0.57
		condition x phase	χ^2^_3_ = 1.853	0.60
		condition x side	χ^2^_3_ = 3.337	0.34
		phase x side	χ^2^_1_ = 0.521	0.47
Markedness	Ordered logistic regression	condition x phase x side	χ^2^_3_ = 0.259	0.97
		condition x phase	χ^2^_3_ = 3.191	0.36
		condition x side	χ^2^_3_ = 1.455	0.69
		phase x side	χ^2^_1_ = 1.923	0.17
Eye white	Generalised linear mixed-effects model	condition x phase x side	χ^2^_3_ = 3.448	0.33
		condition x phase	χ^2^_3_ = 8.33	0.04
		condition x side	χ^2^_3_ = 1.818	0.61
		phase x side	χ^2^_1_ = 1.569	0.21
Number	Linear mixed-effects model	condition x phase x side	F_3, 250_ = 1.101	0.35
		condition x phase	F_3, 42_ = 0.872	0.46
		condition x side	F_3,250_ = 1.185	0.32
		phase x side	F_1,250_ = 0.004	0.95
Angle	Linear mixed-effects model	condition x phase x side	F_3,146_ = 1.287	0.28
		condition x phase	F_3,22_ = 5.922	0.004 *
		condition x side	F_3,146_ = 3.511	0.02
		phase x side	F_1,146_ = 2.189	0.14

^1^ Critical p-value after Bonferroni correction for multiple hypotheses testing: p = 0.0083.

For ‘eye white’ there was a tendency to vary according to our hypothesis, with fewer horses showing eye white in the positive, and more horses showing eye white in the negative treatment phases compared to control phases (χ^2^_3_ = 8.33, p = 0.04). However, the interaction between condition and phase failed to reach significance after Bonferroni correction.

‘Qualitative’ assessment, ‘shape’, ‘markedness’, and the ‘number’ of wrinkles were not affected by any of the fixed effects. Thus, there was neither a significant three-way interaction (condition x phase x side), nor a significant two-way interaction (condition x phase, condition x side, or phase x side, Table 2).

## Discussion

The aim of the present study was to investigate whether variation in the expression of eye wrinkles reflects emotional state in horses. To this end, horses were exposed to distinctively valenced conditions, with the hypothesis that positive conditions would decrease the expression of eye wrinkles whereas negative conditions would increase it. Eye wrinkle expression was assessed using a set of six outcome measures, of which all but ‘shape’ could be assessed reliably. The angle was narrower in the G and wider in the FC condition compared to control phases, indicating relaxation of the inner eye brow raiser during G and contraction of this muscle during FC. Moreover, more horses showed eye white in the PB condition than during the G and FA conditions, whereas in none of these conditions treatment phase significantly differed from the respective control phase. The four other measures did not vary consistently during the four test conditions.

### Intra- and inter-observer reliability

When tested for intra-observer reliability, all six outcome measures could be assessed highly reliably as indicated by the strong agreement between the two scores for all six measures. Almost perfect agreement was also found for the two coders for five of the six measures; only the agreement for ‘shape’ was weaker. We therefore propose to either exclude this measure in future studies or to revise its definition to render it reliable.

In order to arrive at such high reliability, the categorical variables had to be defined more broadly than initially planned, resulting in less fine-grained coding. The disadvantage of this was that ceiling and floor effects were more likely for measures with only three expression levels (‘qualitative’, ‘shape’, and ‘markedness’), and with only two expression levels, ‘eye white’ could only vary in one direction depending on its expression during the control phase. By contrast, the continuous outcome variables allowed a more precise assessment. However, the number of missing values was higher for continuous than for categorical measures, suggesting a need for higher picture quality.

Nevertheless, these measures reflect the type and range of measures by which eye wrinkle expression can be assessed in situ. Given the high intra- and inter-observer reliability, we believe that they may be a useful tool to assess eye wrinkle expression in future studies.

### Assessment of the outcome measures

#### Angle

The angle between the horizontal line through the eyeball and the highest wrinkle was narrower in the G and wider in the FC condition compared to control phases, reflecting relaxation and contraction of the underlying muscle, respectively. Eye wrinkle expression is elicited by the level of contraction of the inner eyebrow raiser, and the angle may therefore be a good indicator of contraction. Contraction of the inner eyebrow raiser has been considered a sign of fear and sadness in humans [18], and in horses the eye wrinkles are more pronounced when the animals are in pain [17]. Interestingly, variation in angle was not only consistent in one negative (FC) but also in one positive condition (G). With the growing interest in the assessment of positive emotional states in animals [22], indicators covering the range from negative to positive emotions are particularly important, as they may not only provide information on the absence of negative emotional states but also on the presence of positive emotional states [35]. However, since results were consistent only for one condition per valence (positive, negative), they do not allow for generalisation to all negative and positive conditions. Thus, it is possible that the angle of eye wrinkles varies depending on discrete emotional states (e.g. those elicited during G and FC) rather than emotional valence in general. Moreover, as discussed in more detail below, we do not have independent evidence that each condition did indeed elicit the expected emotional valence in the horses (in this case considering the FA and the PB condition).

#### Potential reasons why the other outcome measures were not affected

The other five outcome measures did not systematically vary depending on the different conditions. This may simply reflect the fact that these measures were not affected by the horses’ emotional states. However, other explanations should be considered as well.

Firstly, it should be discussed whether the four different conditions the horses were exposed to elicited the expected emotional valence. All conditions were chosen based on literature demonstrating the expected effects on horses’ emotional states. However, since there are as yet no validated measures of emotional valence for horses (hence the present study), we cannot rule out the possibility that the four conditions failed to induce the predicted emotional states in the horses. In particular, the FA condition may have induced frustration while the horses were waiting for the food to arrive instead of a state of positive anticipation [36]. However, the horses were trained to expect the 60 seconds delay before the arrival of the food reward, which should have reduced the likelihood of frustration [36].

Secondly, the duration of the conditions may have affected our results. Emotions are short-term states that are specific to stimuli or events [1]. In contrast, mood states last longer, reflecting the cumulative experience of short-term emotional states, thereby representing past experience [1]. In the present study, we exposed the horses to specific acute conditions hypothesising to induce positive and negative emotional states. It is, however, possible that the duration of the exposure to the four conditions (60 seconds) was not long enough to induce changes in the expression of our outcome measures but that long-term mood could lead to detectable effects. In the horse community, eye wrinkles are thought to be associated with both short-term states, e.g. nervousness and tension [37], as well as long-term states, e.g. depression [38]. As a consequence, future studies should not only investigate the influence of valence and specific conditions but also both the effect of short-term emotional states as well as long-term mood on eye wrinkle expression.

Another possibility is that either emotional state or the associated eye wrinkle expression varied so strongly across the 60 second treatment phases that our pictures, which were selected randomly, did not reflect the induced emotional state. Other possibilities for picture selection would be to include more pictures by sampling at a shorter time interval, or by using independent behavioural indicators of emotion expression as cues to time picture selection to peak emotional state. However, if emotional state or eye wrinkle expression varied so strongly across the 60 second treatment phases that random picture selection did not provide accurate results, then it might be questioned whether the conditions used here were appropriate to study emotion expression or whether eye wrinkle expression can be a reliable indicator of horses’ emotional states.

Thirdly, the categorical variables with only two or three expression levels may have been too constrained, resulting in floor and ceiling effects which may have masked effects of condition and phase. Even the number of eye wrinkles (‘number’), despite being treated as a continuous variable, was rather constrained with a maximum number of six wrinkles and many zeroes, which may have led to floor effects.

Finally, the substantial variability within the four control phases has to be considered. In order to assess the effects of the different conditions on eye wrinkle expression independent of external factors (e.g. time of day, weather condition, and management factors), every treatment phase was paired with a preceding control phase, and the effects were assessed relative to the control phase to which measures during the treatment phase were compared. Variation was relatively high across the four different control phases both between and within horses, suggesting that variation was not only due to individual differences but that individual horses also varied across control phases. Differences between horses were not surprising since individual differences in emotional state are to be expected [39]. Moreover, despite habituation of the horses to the different procedures and the flood lights, the experimental context may have affected them differently. For example, some horses continued to eat straw and did not seem to be affected by the procedure, while others were focusing on the photographers. By contrast, intra-individual differences would have to be explained by changes in emotional state between the different experimental sessions. However, given that intra-individual variation was relatively large, we cannot rule out the possibility that eye wrinkle expression reflects acute and transient emotional states, or that it is sensitive to confounding factors.

### Eye side and laterality

We controlled for eye side (left eye, right eye) by including it as fixed factor in our statistical model. Even though the results were not identical for both eyes, eye side did not significantly affect the outcome measures, providing little indication for laterality effects. There was only a non-significant tendency for an effect of eye side on variation in ‘angle’ across the four conditions but independent of phase. In the literature, visual laterality in response to the valence of an object [40] and in response to the presence of a human [41] have been discussed. Our study was not designed to investigate laterality effects, however, for further validation of ‘angle’ and possibly other measures of eye wrinkle expression as indicators of emotions in horses, it should be examined whether laterality can affect eye wrinkle expression depending on the valence of the conditions the horses are exposed to.

## Conclusion

To our knowledge this was the first study systematically investigating the effect of distinctively valenced conditions on eye wrinkle expression in horses. We established a set of six measures of which five allowed assessing various aspects of eye wrinkle expression reliably. We found that the angle between a horizontal line through the eyeball and the highest wrinkle caused by contraction of the underlying inner eyebrow raiser was consistently affected in two out of the four conditions: G resulted in a narrower angle through muscle relaxation while FC resulted in a wider angle through muscle contraction. The other outcome measures were not affected by the four conditions.

Further research is needed to investigate which aspects of eye wrinkle expression reflect emotional valence in general or are more reflective of specific conditions and thus discrete emotional states. Moreover, it is important to assess how longer lasting conditions affect these measures in view of using them as indicators of emotional well-being in horses.

## Supporting Information

S1 Table Distribution of pictures from the left and the right eye across horses (A) and situations (B).
(DOCX)
