# Supplementary material for: Are Eyes a Mirror of the Soul? What Eye Wrinkles Reveal about a Horse’s Emotional State
Source: PLoS One. 2016 Oct 12;11(10):e0164017. doi: 10.1371/journal.pone.0164017 (PMC5061373; doi:10.1371/journal.pone.0164017)
Supplement: S1 Table — Distribution of pictures from the left and the right eye across horses (A) and situations (B). (DOCX) [file pone.0164017.s001.docx]

S1 Table. Distribution of pictures from the left and the right eye across horses (A) and situations (B).

| **A** | **Horse** | **Left eye** | **Right eye** |
| --- | --- | --- | --- |
|  | 1 | 12 | 16 |
|  | 2 | 24 | 4 |
|  | 3 | 4 | 22 |
|  | 4 | 27 | 0 |
|  | 5 | 12 | 13 |
|  | 6 | 21 | 4 |
|  | 7 | 14 | 4 |
|  | 8 | 10 | 20 |
|  | 9 | 4 | 28 |
|  | 10 | 15 | 16 |
|  | 11 | 0 | 23 |
|  | 12 | 2 | 24 |
|  | 13 | 8 | 13 |
|  | 14 | 0 | 25 |
|  | 15 | 19 | 8 |
|  | 16 | 25 | 0 |
|  | Sum | 197 | 220 |

| **B** | **Situation** | **Left eye** | **Right eye** |
| --- | --- | --- | --- |
|  | G | 58 | 59 |
|  | FA | 28 | 69 |
|  | FC | 60 | 49 |
|  | PB | 51 | 43 |
|  | Sum | 197 | 220 |
